# Supplementary material for: Functional diversity increases the efficacy of phage combinations
Source: Microbiology (Reading). 2021 Dec 1;167(12):001110. doi: 10.1099/mic.0.001110 (PMC8743627; doi:10.1099/mic.0.001110)
Supplement: Supplementary material 1 [file mic-167-1110-s001.pdf]

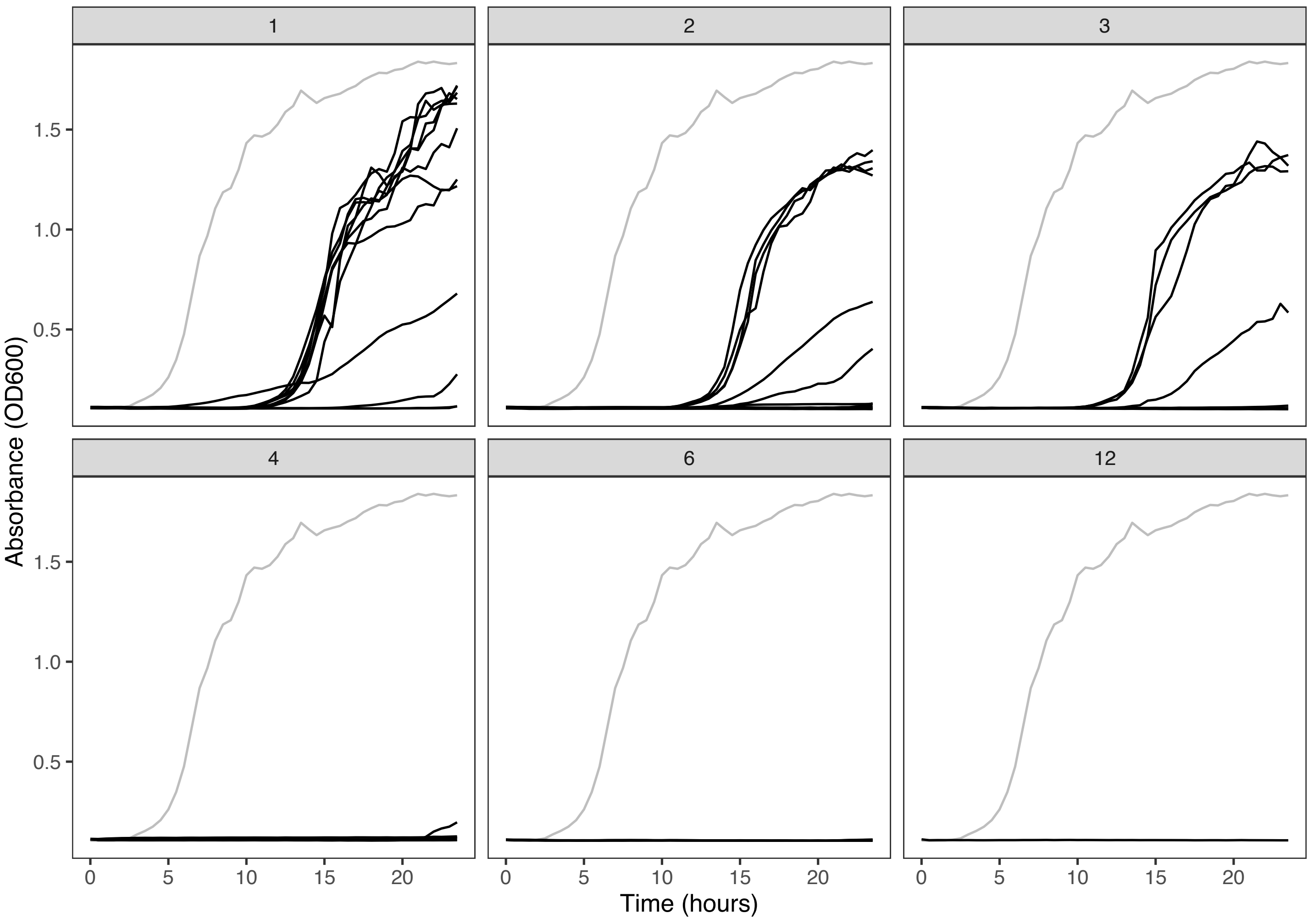

Supplementary Figure 1. Growth of PAO1 in the presence of diverse phage combinations. To assess bacterial growth in the presence of diverse phage combinations, absorbance (OD600) was measured every 30 minutes post phage exposure for 24h. Facet labels indicate the species richness of phage combinations; a random subset of phage combinations at each level of species richness was chosen. The grey line indicates phage-free growth of the bacterial host.

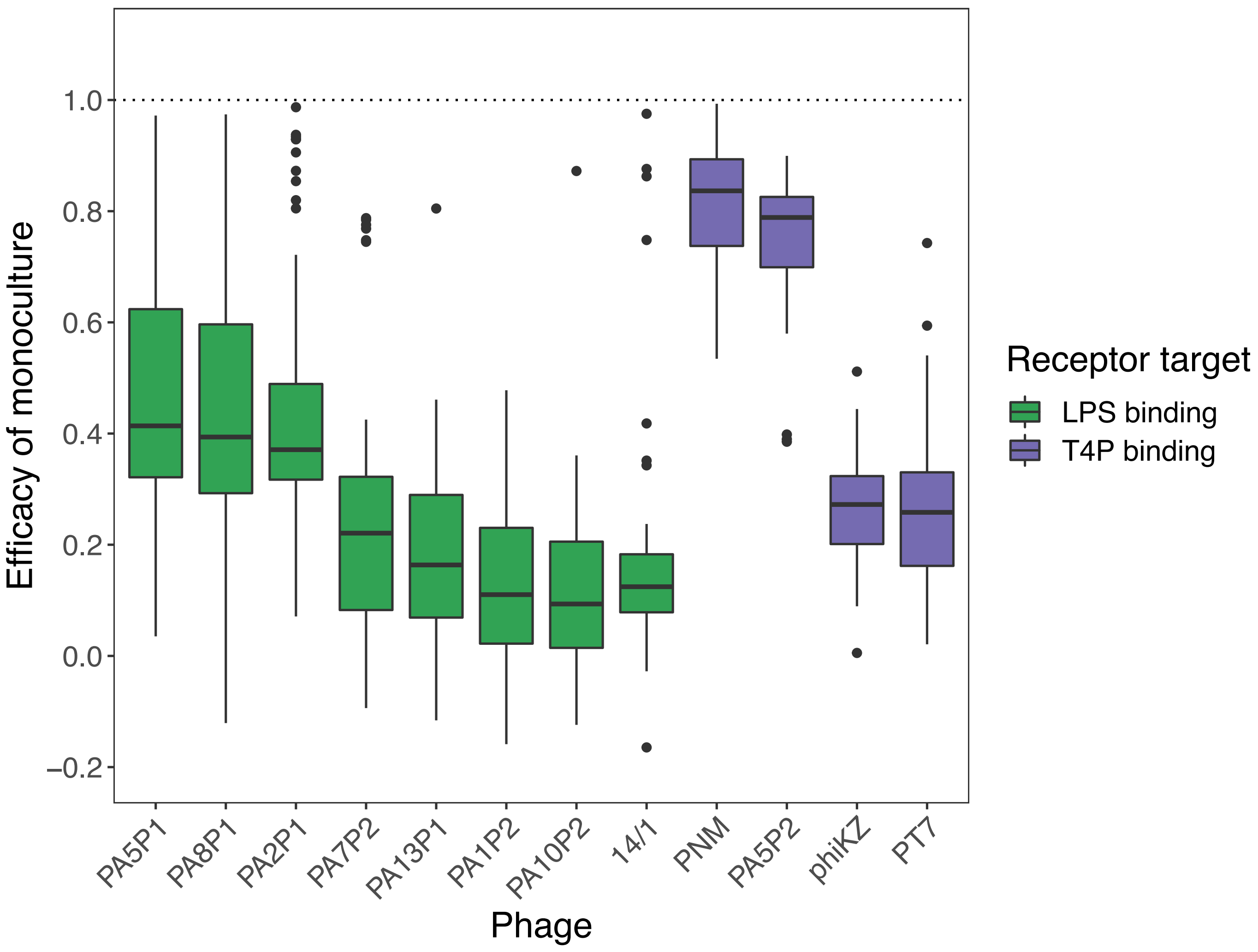

Supplementary Figure 2. Efficacy of phage monocultures. Boxplots showing the distribution of Efficacy, measured as suppression of bacterial growth by phages relative to phage-free populations, for monocultures of all phages included in this study. The dashed line indicates the theoretical maximum reduction (*i.e.*, no bacterial growth detected).
